# Connecting the Dots Between the Gut–IGF-1–Prostate Axis: A Role of IGF-1 in Prostate Carcinogenesis

**DOI:** 10.3389/fendo.2022.852382

**Published:** 2022-03-15

**Authors:** Makoto Matsushita, Kazutoshi Fujita, Koji Hatano, Marco A. De Velasco, Hirotsugu Uemura, Norio Nonomura

**Affiliations:** ^1^Department of Urology, Graduate School of Medicine, Osaka University, Suita, Japan; ^2^Department of Urology, Faculty of Medicine, Kindai University, Osakasayama, Japan; ^3^Department of Genome Biology, Faculty of Medicine, Kindai University, Osakasayama, Japan

**Keywords:** prostate cancer, IGF-1, short-chain fatty acids, gut microbiome, bacteria

## Abstract

Prostate cancer (PCa) is the most common malignancy in men worldwide, thus developing effective prevention strategies remain a critical challenge. Insulin-like growth factor 1 (IGF-1) is produced mainly in the liver by growth hormone signaling and is necessary for normal physical growth. However, several studies have shown an association between increased levels of circulating IGF-1 and the risk of developing solid malignancies, including PCa. Because the IGF-1 receptor is overexpressed in PCa, IGF-1 can accelerate PCa growth by activating phosphoinositide 3-kinase and mitogen-activated protein kinase, or increasing sex hormone sensitivity. Short-chain fatty acids (SCFAs) are beneficial gut microbial metabolites, mainly because of their anti-inflammatory effects. However, we have demonstrated that gut microbiota-derived SCFAs increase the production of IGF-1 in the liver and prostate. This promotes the progression of PCa by the activation of IGF-1 receptor downstream signaling. In addition, the relative abundance of SCFA-producing bacteria, such as *Alistipes*, are increased in gut microbiomes of patients with high-grade PCa. IGF-1 production is therefore affected by the gut microbiome, dietary habits, and genetic background, and may play a central role in prostate carcinogenesis. The pro-tumor effects of bacteria and diet-derived metabolites might be potentially countered through dietary regimens and supplements. The specific diets or supplements that are effective are unclear. Further research into the “Gut–IGF-1–Prostate Axis” may help discover optimal diets and nutritional supplements that could be implemented for prevention of PCa.

## Introduction

Prostate cancer (PCa) is the most common malignancy in men worldwide and the fifth most common cause of cancer-related death with as many as 360,000 men dying of PCa annually (1). PCa morbidity varies somewhat by region and race, and has consistently been increasing in recent years (2, 3). Although androgen deprivation therapy is very effective for PCa, high-grade PCa becomes androgen resistant, which makes subsequent treatment challenging. Therefore, it is important to find new targets for the prevention and treatment of high-risk PCa.

The age-adjusted prevalence of latent PCa at autopsy in Japanese migrants in Hawaii >50 years old was higher than that of Japanese men living in Japan (25.6% vs. 20.5%), suggesting that PCa risk is not only altered by genetic factors but also by various environmental factors, such as diet (4). Many studies have reported that excessive intake of animal fat, carbohydrate, and dairy products increases PCa risk. However, different cohorts have yielded different results, and no consensus has been reached (5). This is because diet affects PCa development and progression through multiple mechanisms (6). The relationship between the diet and PCa is complex and not fully understood and as a result has hindered PCa prevention and treatment strategies *via* dietary interventions.

We recently identified a novel mechanism by which specific intestinal bacteria promote PCa through insulin-like growth factor I (IGF-1) signaling (7). Various studies have shown that diet can disrupt gut microbial composition resulting in dysbiosis and loss of homeostasis, affecting local intestinal disease as well as diseases and disorders in distant organs, such as the liver and brain (8–13). These relationships have been referred to as gut–liver axis and gut–brain axis, respectively. In cancer biology, the relationships between gut microbiota and various types of cancer, such as colorectal, hepatocellular, and breast cancer, have been well studied. In contrast, little is known about the influence of the gut microbiota on PCa (14). IGF-1 is implicated in the pathogenesis of PCa and may be the key player that links diet to prostate carcinogenesis and progression that is mediated by gut microbes. This review summarizes the present knowledge of the functions of IGF-1 in PCa progression, especially its relationship to diet and gut microbiota.

## Mechanism of IGF-1 Signaling

IGF-1 is a growth factor that plays a crucial role in cell proliferation and physical growth. IGF-1 signal transduction is mediated through the IGF-1 receptor (IGF1R) and insulin receptor (INSR). The structures of IGF1R and INSR are highly homologous (15). These receptors stimulated by IGF-1 activate tyrosine kinase activity directed at the β subunit, resulting in substrate phosphorylation, such as insulin receptor substrate (IRS) 1, IRS2, and Src homology collagen. The phosphorylated residues are recognized by the signaling molecules p85 and Grb2, which stimulate the phosphoinositide 3-kinase (PI3K) and mitogen-activated protein kinase (MAPK) signaling cascades. These signaling cascades mediate crucial biological functions of IGF-1 (15).

IGF-1 production in the healthy liver is mainly positively regulated by growth hormone (GH) signaling through the GH receptor pathway (16, 17). IGF-1 is released into circulation. IGF-1 is produced in other organs by various other cells that express IGF1R and is activated *via* autocrine signaling, although hepatocytes do not express enough IGF1R (18). Therefore, in liver-specific IGF-1-deficient mouse models, circulating IGF-1 levels are reduced to <20% of control mice and GH levels are elevated, but physical growth is normal (19). The bioavailability of circulating IGF-1 is regulated by the IGF-binding protein (IGFBP) family that blocks access to its receptor (20). In particular, IGFBP3 produced by Kupffer cells in the liver is important in IGF-1 homeostasis (21).

## Effects of IGF-1 on Organs

IGF-1 has tissue-specific roles through IGF1R and INSR. Muscle-specific IGF1R knockout mice display disrupted muscle fiber formation and reduced muscle weight early in development (22). However, muscle weight of differentiated muscles is not affected in mice with IGF1R knockout (23). IGF-1 has a significant effect on physical growth, but not on adult physiology. IGF-1 is also critical in bone growth and maintenance during postnatal life. IGF-1 can directly affect chondrocytes and osteoblasts and increase ephrin ligand-receptor signaling, leading to the differentiation of each cell. IGF-1 signaling also inhibits the formation of differentiated osteoclasts, contributing to bone growth (24). In the pancreas, IGF-1 signaling retains normal β-cell function, which is necessary to maintain glucose tolerance *in vivo* (25, 26). *In vitro*, IGF-1 stimulates expression of cellular communication network factor 5 (CCN5) and promotes β-cell proliferation (27). The phenotype of diabetes by blocking IGF-1 signaling is more obvious in mouse models lacking both IGF1R and INSR in β-cells (28). IGF-1 signaling is required for adipocyte development and function in adipose tissue, which is a major nutrient storage site. Mice lacking IGF1R and INSR in adipocytes contain almost no adipose tissue and develop significant diabetes, dyslipidemia, and fatty liver (29). IGF-1 is involved in myeloid cell function. IGF-1 activates M2 macrophages. Secretion of IGF-1 by the macrophages in turn leads to insulin resistance in mice fed a high-fat diet (30). A very important role of IGF-1 is its effect on the endocrine system. IGF-1 can directly support thyroid hormone production, and organ-specific IGF-1 signal loss reduces thyroid hormone and significantly increases thyroid stimulating hormone (TSH) levels (31). IGF1R and INSR knockout inhibit the development of the adrenal cortex and testes, and reduce testosterone levels. How IGF-1 signaling affects adrenal and testes function remains unknown (32). The role of IGF-1 signaling in prostate development and normal prostate physiology has not been established *in vivo*, however, silencing IGF-1 in not only the WPMY-1 prostate stroma cell line, and but also BPH-1, a prostate epithelium cell line, decreased cell proliferation and increased apoptosis rate *in vitro* (33). In human, *IGF1R* is located on the long arm of chromosome 15, and 36 different probable mutations have been reported (34). Most patients are heterozygous carriers, and all show pre- and postnatal growth retardation and dysmorphic features, such as a triangular face. The collective findings reveal that IGF-1 is an essential hormone for normal growth and maintain homeostasis.

## Cancers and IGF-1 Signaling

IGF-1 is involved in several diseases. It is clear that diabetes is influenced by IGF-1 because of its effect on pancreatic β-cell function. IGF-1 increases nutrient-stimulated insulin release. The increased level of insulin increases IGF-1 production by stimulating GH signaling (35). Impaired insulin secretion due to type 1 diabetes lowers serum IGF-1 levels, and improves glycemic control in patients with type 2 diabetes leads to increased IGF-1 levels (36–38). Obese individuals have lower serum IGF-1 levels than normal-weight individuals, although over nourishment is associated with high insulin and IGF-1 levels (39). Several large studies found that serum IGF-1 levels are highest in both men and women with a body mass index of 24–27 kg/m^2^ (40, 41). A possible reason why serum IGF-1 levels are decreased in obese patients is that increased free IGF-1 fraction by reduction in IGFBP production enhances negative feedback on GH secretion by the pituitary gland (42). Therefore, IGF-1 bioactivity may not be decreased, even in obese patients. In a study of 27 samples of benign prostatic hyperplasia (BPH) patients, there was no significant relationship between serum IGF-1 levels and prostate volume (P = 0.91). However, the gene expression of IGF-1 in prostate tissue was significantly increased (P = 0.001) and the expression of IGFBP3 was significantly decreased (P = 0.003) in patients with larger prostate size (>30 mL) (43). Local IGF-1 was reportedly upregulated in hyperplastic prostate tissues (33). Patients with acromegaly characterized by GH hypersecretion display high IGF-1 levels, and acromegaly patients are highly susceptible to IGF-1 related diseases including diabetes mellitus and BPH, suggesting IGF-1 regulation of various diseases. Acromegaly patients <40 years of age were found to have significantly larger prostate than healthy men (18.2 vs. 28.5 mL, P < 0.001), and suppression of GH and IGF-1 using octreotide caused prostate shrinkage (44).

IGF-1 has been associated with the development and progression of some cancer types due to its function in activating the MAPK and PI3K signaling pathways (15). A positive association was observed between serum IGF-1 level and overall cancer risk in men in the United Kingdom (hazard ratio [HR] = 1.03 per 5-nmol/L increment in IGF-1) and specific cancer risk, such as prostate, melanoma, kidney, and thyroid (HR = 1.09, 1.08, 1.10, and 1.22, respectively) (45). In these cancer types, basic studies have also shown an association with IGF-1 signaling (46–48). Although melanoma cells do not produce IGF-1, activation of the MAPK and PI3K signaling pathway by paracrine stimulation of IGF-1 from stromal fibroblasts enhanced survival, migration, and growth of melanoma cells only from biologically early tumors (46). A cell line derived from metastatic clear cell renal cell carcinoma highly expresses IGFBP3 and IGF-1 compared to normal proximal tubule cell, and the autocrine actions of IGF-1 and IGFBP3 promote and inhibit cell proliferation, respectively (47). IGF-1 secreted by M2-like tumor−associated macrophages promote the invasion and stemness of C643 cells, an anaplastic thyroid carcinoma cell line, by activating PI3K signaling (48). Furthermore, IGF-1 is involved in bone metastasis biology, such as in homing, dormancy, colonization, and expansion (49). In an *in vivo* study, the presence of high IGF-1 levels in the primary tumor environment tended to induce cancer cells to metastasize to bone, and cancer cell lines that highly expressed IGF1R were prone to display enlarged bone mass (50, 51). IGF1R is highly expressed in PCa cells. Therefore, PCa may be susceptible to IGF-1 signaling (52). The relationship between IGF-1 and PCa is detailed in the next section.

## Role of IGF-1 Signaling in Prostate Cancer Biology

**Figure 1:**
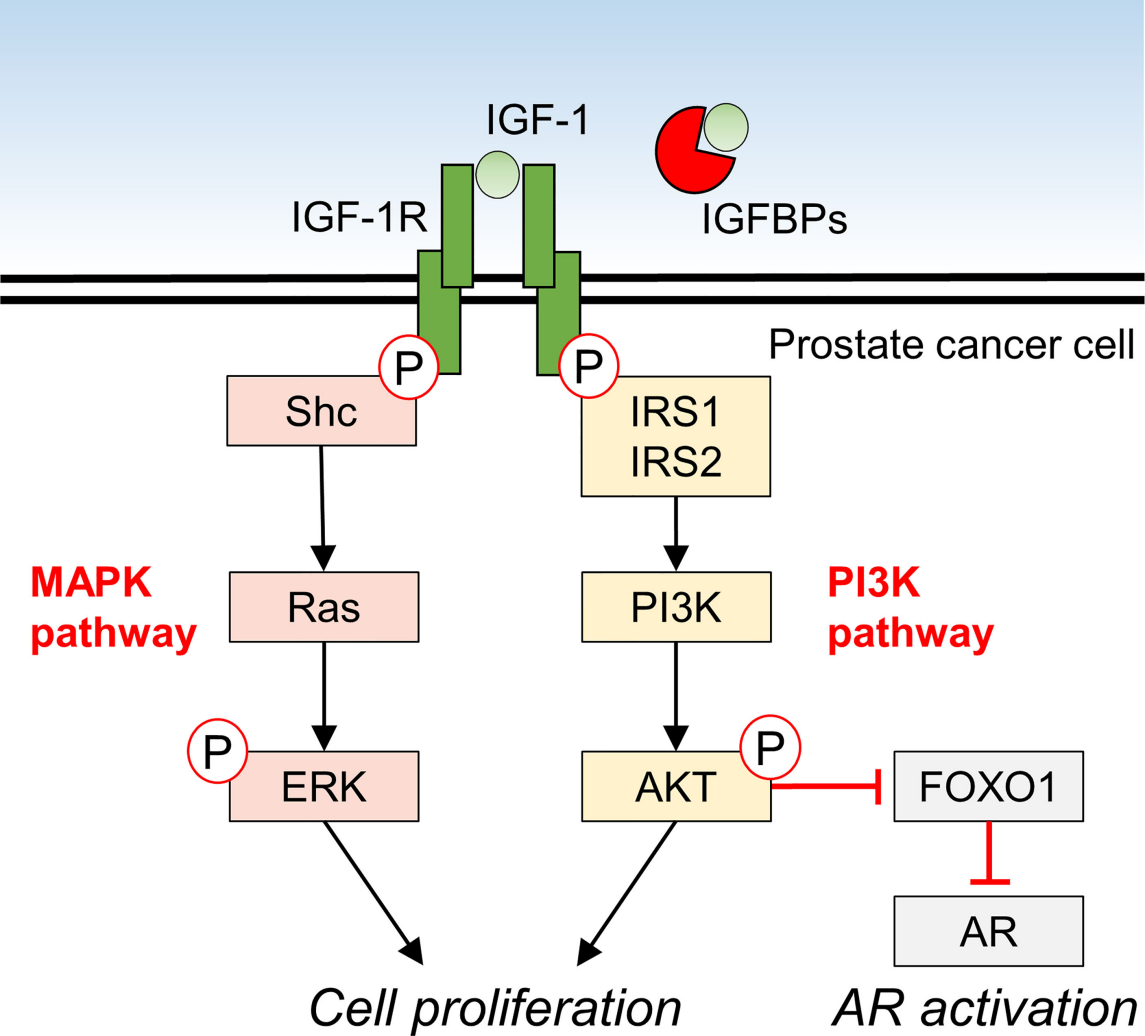
Molecular mechanism of IGF-1 signaling and downstream effects in prostate cancer cells.

IGF-1 promotes the proliferation of 22RV1 and DU145 PCa cell lines *in vitro* (7). In these cell lines, protein kinase B (AKT) in the PI3K pathway and extracellular signal-regulated kinase (ERK) in the MAPK pathway were phosphorylated in an IGF-1 dose dependent manner, suggesting that IGF-1 directly influences PCa proliferation (7). *In vivo*, IGF-1 expression was reportedly reduced in xenografts of Los Angeles PCa-4 (LAPC-4) in mice fed a low-fat diet, and tumor volume was suppressed (53). IGF-1 decreased miR-143 expression and increased IGF1R expression in PC-3 and DU145 cells, and made these cell lines more resistant to docetaxel treatment, suggesting that IGF-1 levels are also involved in resistance to treatment in PCa (54). IGF-1 is also implicated in castration-resistant PCa and has been shown to activate androgen receptor (AR) signaling in prostate cancer cells *via* the IGF-1R-forkhead box protein O1 (FOXO1) signaling axis (**Figure 1**) (55–57).

Elevated blood IGF-1 levels increase the future risk of PCa in healthy men (45). Acromegaly patients with systemically high GH and IGF-1 levels also have significantly higher incidence of PCa and risk of PCa-related mortality (HR = 1.33 and 1.44, respectively), suggesting that IGF-1 has a positive effect on PCa development and progression, even in humans (58). Several studies reported that blood IGF-1 levels in elderly men with suspected PCa on screening tests are not associated with cancer positivity (59, 60). Serum IGF-1 levels in 94 men who required prostate biopsy showed no significant difference between positive and negative cancer (26.4 vs. 23.7 nmol/L; P = 0.08) (59). This discrepancy suggests that prostate epithelial cells may be at an increased risk of cancer development or progression only after prolonged exposure to high concentrations of IGF-1. Suppression of IGF-1 signaling is a potential therapeutic approach, because the IGF1R inhibitor in combination with castration inhibited PCa growth in rodent models of bone metastasis and subcutaneous xenografts (61, 62). However, in a phase 2 study, limsitinib, the most extensively evaluated IGF1R inhibitor, failed to significantly improve levels of prostate-specific antigen after 12 weeks of treatment and did not improve overall survival in men with metastatic castrate-resistant PCa (63). In the future, as a more potent treatment strategy, a combination of novel IGF1R inhibitors and existing prostate cancer therapies is expected to be effective.

## SCFAs as Major Metabolites of Intestinal Bacteria

In recent years, studies investigating the interactions between gut microbiota and its host has focused on recognizing an essential factor that influences homeostasis. One of the mechanisms by which intestinal bacteria affect humans is through bacterial structural components and their metabolites. Short-chain fatty acids (SCFAs) are major bacterial metabolites that play an important role in physiology. SCFAs include fatty acids with six or fewer carbon atoms. Of these, acetate (C2), propionate (C3), and butyrate (C4) are mainly produced by fermentation of dietary fiber by intestinal bacteria (64). Bacterial-derived SCFAs affect not only locally the gut but also distant organs in various ways. The anti-inflammatory effect is one of the major characteristics of SCFAs. Bacterial-derived butyrate promotes the differentiation of colonic regulatory T cells, suppresses inappropriate mucosal immunity, and improves local colitis and distant arthritis (65, 66). Propionic acids that reach the liver *via* the portal circulation increase glycogen synthesis and storage, improve insulin sensitivity, and repress lipogenesis in hepatocytes, resulting in the maintenance of energy homeostasis (67, 68). In the central nervous system (CNS), bacterial-derived SCFAs contribute to normal maturation of microglia *via* the free fatty acid receptor 2 (FFAR2) as a SCFA receptor (69). SCFAs also have some effects on several brain functions involved in circadian rhythm and appetite control (70).

SCFAs are also involved in the development and progression of cancer (71). In colorectal cancer, SCFAs, especially butyrate, function as anti-inflammatory metabolites and histone deacetylate (HDAC) inhibitors, resulting in the suppression of cancer progression (72). HDAC is a very important enzyme that modulates the expression of genes involved in signaling pathways, such as MAPK and Wnt (72). Therefore, accumulation of butyrate in some types of cancer cells that abundantly express a variety of HDACs, could inhibit their proliferation and promote apoptosis. In PCa, HDAC1, 2, and 3 are highly expressed (73). *In vitro*, sodium butyrate can directly decrease androgen receptor gene expression in LNCaP and C4-2 PCa cells, and can decrease the viability of these cells at concentrations >2.5 mM (74). However, it was demonstrated that high concentrations of butyrate were needed to inhibit colorectal cancer growth as an HDAC inhibitor, while <5 mM butyrate promoted cancer growth (75). SCFAs metabolized by intestinal bacteria are absorbed into the portal circulation and reach the liver, where most of the SCFAs are consumed (76). Therefore, only a small amount of bacteria-derived SCFAs can reach the prostate *via* systemic circulation. *In vivo*, bacteria-derived butyrate is not likely to work as an HDAC inhibitor in PCa due to this low concentration.

## IGF-1 Mediated Effect of SCFAs on Prostate Cancer Proliferation

In young mice, gut microbiota-derived SCFAs are likely to induce IGF-1 production, suggesting that SCFAs modulate the bone and physical growth (77). The authors also described that germ-free mice and mice orally administered antibiotics showed lower cecal SCFA concentrations and IGF-1 production, resulting in decreased bone growth (77). SCFAs play a positive role in bone formation *via* an IGF-1-mediated mechanism. Unfortunately, the pathway by which SCFAs result in the elevation of IGF-1 is still not well understood.

We have reported that SCFAs metabolized by intestinal bacteria contribute to PCa growth by increasing systemic and prostate local IGF-1 productions, and revealed the “gut–prostate axis” involving bacterial metabolites (7). Prostate-specific phosphatase and tensin homolog (*PTEN*)*-*knockout mice [*Pb*-Cre+; *Pten*^fl/fl^] were used as a PCa model. In these mice, a western-style high-fat diet (HFD) containing mainly lard accelerated PCa growth (78). This diet-induced PCa growth was inhibited by oral administration of metformin or celecoxib, as well as by an antibiotic mixture (ampicillin, vancomycin, neomycin, and metronidazole) (7, 78, 79). Antibiotics cause substantial changes in the composition of the gut microbiota of HFD-fed mice. Fecal SCFAs in the mice were reportedly reduced by 75%, resulting in decreased production of IGF-1 in the liver and prostate. In addition, phosphorylation of IGF-1R, ERK, and AKT was reduced in PCa cells of mice fed a HFD who received antibiotic, suggesting that decreased IGF-1 might suppress the activity of MAPK and PI3K signaling cascades. Oral supplementation of SCFAs to mice fed a HFD who received antibiotic resulted in increased serum IGF-1 levels and promoted prostate cancer growth. These results suggest that SCFAs derived from intestinal bacteria promote PCa growth through IGF-1 signaling, although butyrate in SCFAs may inhibit cancer cell proliferation as an HDAC inhibitor.

The examination of mice treated with antibiotics has revealed the absence of members of the family Rikenellaceae, order Clostridiales in the gut microbiota. Examination of the gut microbiota of men with a high-risk of PCa has revealed the increased abundance of genus *Alistipes* belonging to Rikenellaceae and the genus *Lachnospira* compared to men at low risk of PCa and those who are PCa-free (80). These bacterial taxa are associated with SCFA content in the stool and are SCFA-producing bacteria (81–86). These results suggest that SCFAs and their producing bacteria in the gut may be risk factors for PCa in humans and mice. Consumption of milk and other dairy products increase the dietary intake of SCFAs, thus resulting in the increase of serum IGF-1 levels and that is because these are the among the few foods that contain butyrate (87, 88). Many epidemiological studies have indicated that consumption of milk and dairy products increases the risk of PCa (5). This increased risk may be due to the butyrate contained in these foods (88). While dairy products are essential for nutrition and may a preventive effect in various diseases, including colorectal cancer (89), the roles are complex and most likely context-dependent. For example, low-fat milk containing no SCFAs does not increase the risk of PCa, unlike whole milk (90). Additional studies have reinforced this notion. In the NIH-AACR Diet and Health Study, during 7 years of follow-up, the highest quintile of dairy food intake had a significantly lower risk of colorectal cancer (relative risk [RR] = 0.85, P = 0.01) and a higher risk of prostate cancer (RR = 1.06, P = 0.01) compared to the lowest quintile (91). We hypothesize that the regulation of IGF-1 signaling contributing to prostate cancer risk in a real-world setting is increased by intestinal factors.

There are still some questions that need to be clarified regarding the gut–prostate axis involving SCFAs and IGF-1 signaling. It has been also reported that butyrate and propionate may have inhibitory effects on prostate cancer (74, 92). In our animal study, we found that a mixture of SCFAs (acetate, propionate, and butyrate) promoted prostate cancer growth (7), but we have not been able to determine which types of SCFAs are responsible for this promotive effect and at what concentration. Perhaps these may act cooperatively. Furthermore, although several G protein-coupled receptors, such as GPR41 and GPR43, are known as SCFA receptors, neither the receptor nor the signaling pathway(s) involved in the regulation of IGF-1 by SCFAs have been established (93, 94). Finally, the impact of interventions on the gut–prostate axis in human using fecal microbiota transplantation (FMT) or pro/prebiotics has not been studied. There are however several basic studies that have reported that FMT derived from prostate cancer individuals altered prostate cancer progression in mouse (95, 96), and we think that this axis may be a promising therapeutic target.

## Conclusion

**Figure 2:**
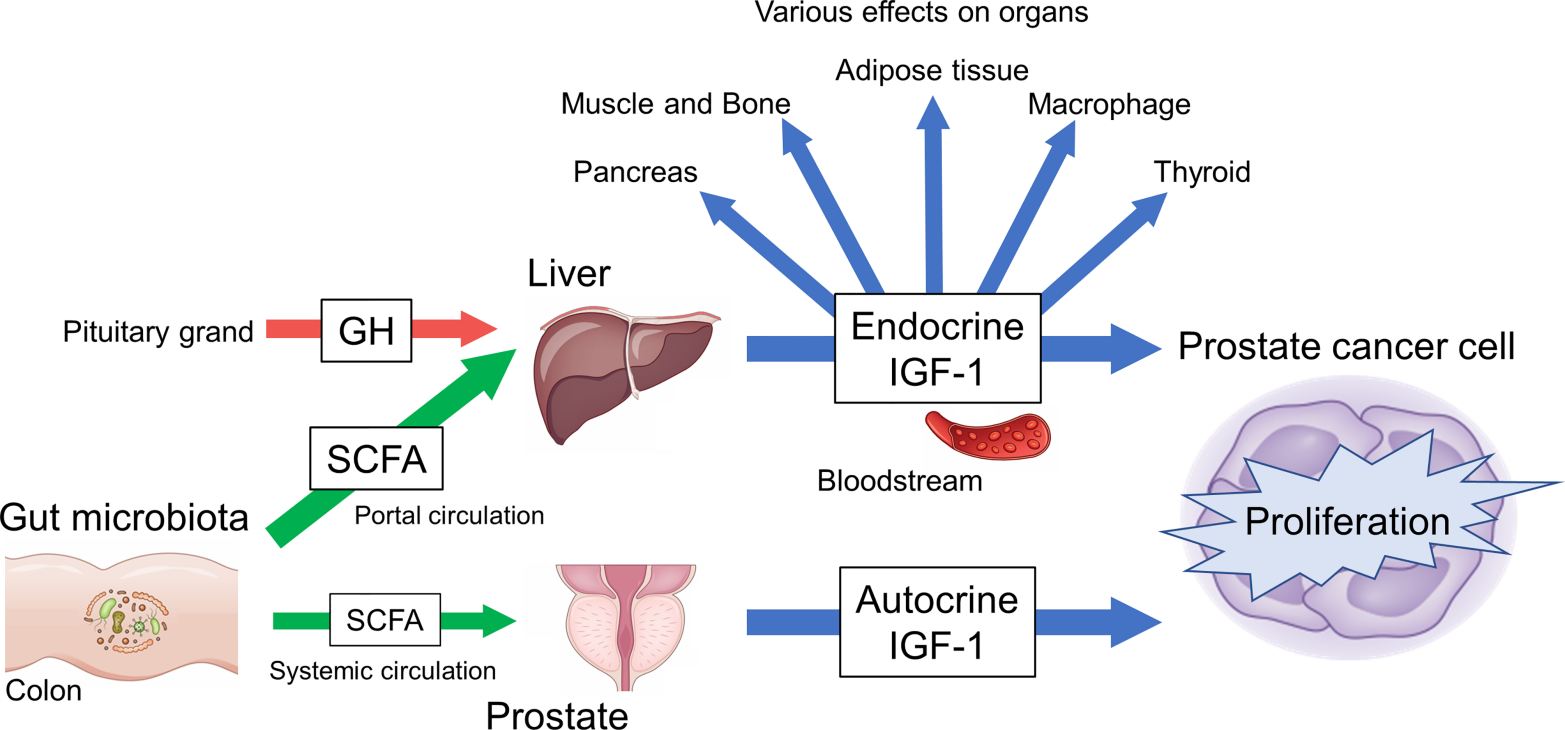
Overview of gut–IGF-1–prostate axis mediated by gut microbiota-derived SCFAs.

IGF-1 is an essential hormone for physical growth and has various effects in several diseases, especially prostate cancer, where it functions as an exacerbating factor. *In vivo*, local and systemic IGF-1 production might be regulated by SCFAs, which is in turn influenced by gut factors, such as gut microbiota and diet. The data thus far indicate that the gut–IGF-1–prostate axis is connected by SCFAs (**Figure 2**). This axis could provide a new direction for effective PCa treatment and prevention strategies. However, there are factors that remain unclear such as detailed mechanisms of IGF-1 regulation by SCFAs and the continuous control of SCFA levels in humans. Further study of the gut–IGF-1–prostate axis is needed to provide additional answers.

## Author Contributions

MM: writing—original draft preparation. KF, KH, and MV: conceptualization and writing—review and editing. HU and NN: supervision. All authors have read and agreed to the published version of the manuscript.

## Funding

This work was supported by JSPS KAKENHI Grant Number JP21K09421.

## Conflict of Interest

The authors declare that the research was conducted in the absence of any commercial or financial relationships that could be construed as a potential conflict of interest.

## Publisher’s Note

All claims expressed in this article are solely those of the authors and do not necessarily represent those of their affiliated organizations, or those of the publisher, the editors and the reviewers. Any product that may be evaluated in this article, or claim that may be made by its manufacturer, is not guaranteed or endorsed by the publisher.
